# Music-based casual video game training alleviates symptoms of subthreshold depression

**DOI:** 10.3389/fpubh.2022.961425

**Published:** 2022-08-03

**Authors:** Ximeng Li, Moyi Zheng, Yuchang Zhang, Yueyun Wang, Lu Nie, Yuan Yuan, Tianyi Qian, Yixuan Ku

**Affiliations:** ^1^Center for Brain and Mental Wellbeing, Department of Psychology, Sun Yat-sen University, Guangzhou, China; ^2^School of Art, Sun Yat-sen University, Guangzhou, China; ^3^Tencent Healthcare, Shenzhen, China; ^4^Peng Cheng Laboratory, Shenzhen, China

**Keywords:** music-based video game, subthreshold depression, intervention, anxiety, stress, self-efficacy

## Abstract

**Objectives:**

In this preregistered study, we investigated the beneficial effects of music-based casual video game training on the depression, anxiety and stress symptoms in a cohort of young individuals with subthreshold depression and the underlying mechanisms.

**Methods:**

The study included 56 young individuals (18–26 years of age) with subthreshold or mild depression based on the Beck Depression Inventory-II (BDI-II) scores between 14 and 19. They were randomly assigned into the experimental group (*n* = 28) or the control group (*n* = 28). The experimental group underwent music-based casual video game training for 4 weeks. During the same time, the control group participants conducted daily life activities without any intervention. The study participants in the two groups were analyzed using the Depression Anxiety and Stress Scale (DASS-21) during the baseline before the intervention, as well as DASS-21, Positive and negative Affect Scale (PANAS), General Self-efficacy Scale (GSES), and the Emotional Regulation Questionnaire (ERQ) twice a week during the 4 weeks of intervention.

**Results:**

The depression, anxiety, and stress symptoms were significantly reduced in the experimental group participants after 4 weeks of music-based video game training compared with the control group. The DAS scores in the experimental group were alleviated in the third and fourth weeks of training compared with the control group. Moreover, analysis using the general linear model demonstrated that the number of training weeks and self-efficacy were associated with significant reduction in depression, anxiety and stress. Furthermore, our results demonstrated that self-efficacy was correlated with positive emotion and emotional regulation.

**Conclusion:**

Our study showed that music-based casual video game training significantly decreased depression, anxiety, and stress in the young individuals with subthreshold depression by enhancing self-efficacy.

## Introduction

Depression is a mood disorder characterized by persistent feeling of sadness, loss of interest in the daily activities, poor emotional regulation, and suicidal tendencies. Depression includes many different states that range from absence of any depression to very severe depression (1, 2). Patients with subthreshold depression show clinically significant symptoms of depression but do not meet the criteria for positive diagnosis of a major depressive disorder. However, subthreshold or mild depression is a risk factor for major depression (3). Furthermore, subthreshold or mild depression is highly prevalent (4) and is associated with significant economic burden, functional impairment, and suicide risk (5). Therefore, identification and intervention of subthreshold or mild depression is essential for preventing the onset of major depression and other adverse outcomes.

Traditional treatments for depression such as cognitive behavioral therapy (CBT), interpersonal psychotherapy (IPT), and supportive therapy are also commonly used to treat subthreshold or mild depression symptoms. These interventions demonstrate low to moderate effects in the youth (2). In recent years, several digital interventions including video game training have emerged for treating depression especially in the youth (6). Video game interventions have several advantages over traditional treatments for depression. Firstly, therapy with video games can be engaging and acceptable to individuals who find CBT to be boring and do not recognize or accept their mental health problem (7). Secondly, face-to-face CBT is conducted with a therapist on a one-to-one basis or in groups with other individuals needing similar help. However, video game training can be personalized to the needs of every patient at a lower cost (8). A recent systematic review demonstrated that the video game-based interventions such as serious games (9), commercial video games (10), and casual video games (11) significantly reduced the symptoms of depression (9). Moreover, casual video games are easy, interesting, brief and convenient, and do not require additional knowledge or skills of operation (11). A systematic review demonstrated that casual video games significantly reduced the symptoms of anxiety and depression, and improved the mood (11).

The video game-based interventions reduce the symptoms of anxiety and depression by modulating emotion and cognition. The Casual Video Games were defined as fun, fast to access, and simple to learn and requiring no previous video game skills or time commitment to play [Casual Games (12)]. Casual video games decrease the mental distress by providing participants with a relaxing and entertaining experience (13). Video game interventions also modulate emotions *via* the cognition pathway. A meta-analysis of several studies showed that frequent playing of video games moderately enhanced the executive functions (14). Another study demonstrated that action video game training improved the executive functions (15). Furthermore, executive functions positively regulated emotions, enhanced performance, and positive affect (16). A meta-analysis of several studies demonstrated that working memory training in executive functions enhanced emotional regulation *via* changes in the electrical and physiological activities of the brain (17). Effective regulation of emotions is essential for maintaining normal social functions as well as the physical and mental health of an individual. On the contrary, excessive emotional responses, individual differences in executive function, and abnormal regulation of the brain emotion networks are associated with abnormal social behaviors including anxiety disorders and depression (18). These data suggested that training of executive functions may alleviate emotional problems. A previous report demonstrated that improvements in cognitive control and executive functions by training significantly reduced the depressive symptoms characterized by intrusive thinking (19).

Video game interventions may also influence the mood by increasing self-efficacy, which refers to the belief that a person is capable of coping with stressful events or challenges (20). High self-efficacy is associated with lower levels of negative emotions and higher levels of positive emotions and achievements (21). Individuals with higher self-efficacy show a sense of control in difficult situations and believe that they can find a solution to their problems (22). The video game settings provide intermittent reinforcement and optimism for the players in a school or a working environment (7). Individuals with higher self-efficacy show a higher rate of behavioral initiative, persistence, and positive emotions because of their belief that good outcomes can be achieved through positive behaviors (23, 24). Therefore, video games may strengthen the perception of self-efficacy among the players and enable them to solve problems in their daily life by increasing positive emotions.

Several studies have shown that music therapy and other music-based interventions reduce the depressive symptoms in the adults (25–27). Furthermore, music therapy and other music-based interventions decrease depression in adolescents and elderly patients by reducing internalization problem, anxiety and depression, and facilitating their psychological needs (28–30). Specifically, music-based interventions effectively reduce internalization problems in children and adolescents (28). They also effectively reduce anxiety and depression levels in the adolescents (29) and contribute to the psychological needs of the elderly (30). Music therapy directly affects the emotional response and improves the ability of the patients to regulate emotions. Music activates the brain regions associated with reward and cognitive control, including the anterior cingulate cortex (ACC), orbitofrontal cortex (OFC) and lateral prefrontal cortex (PFC), and suppresses activity in the amygdala (31–34). These results are consistent with the activation patterns of the emotional regulation process. A recent study of adolescents with depression showed significant reduction in the depressive symptoms and significant improvement in mood regulation after 12 sessions of music therapy (35).

In the present study, we investigated the potentially beneficial effects of music-based casual video game training for 4 weeks in young individuals with subthreshold or mild depression and the underlying mechanisms. The study protocols and predictions were pre-registered on AsPredicted.org (#80788).

## Materials and methods

### Participants

**Figure 1:**
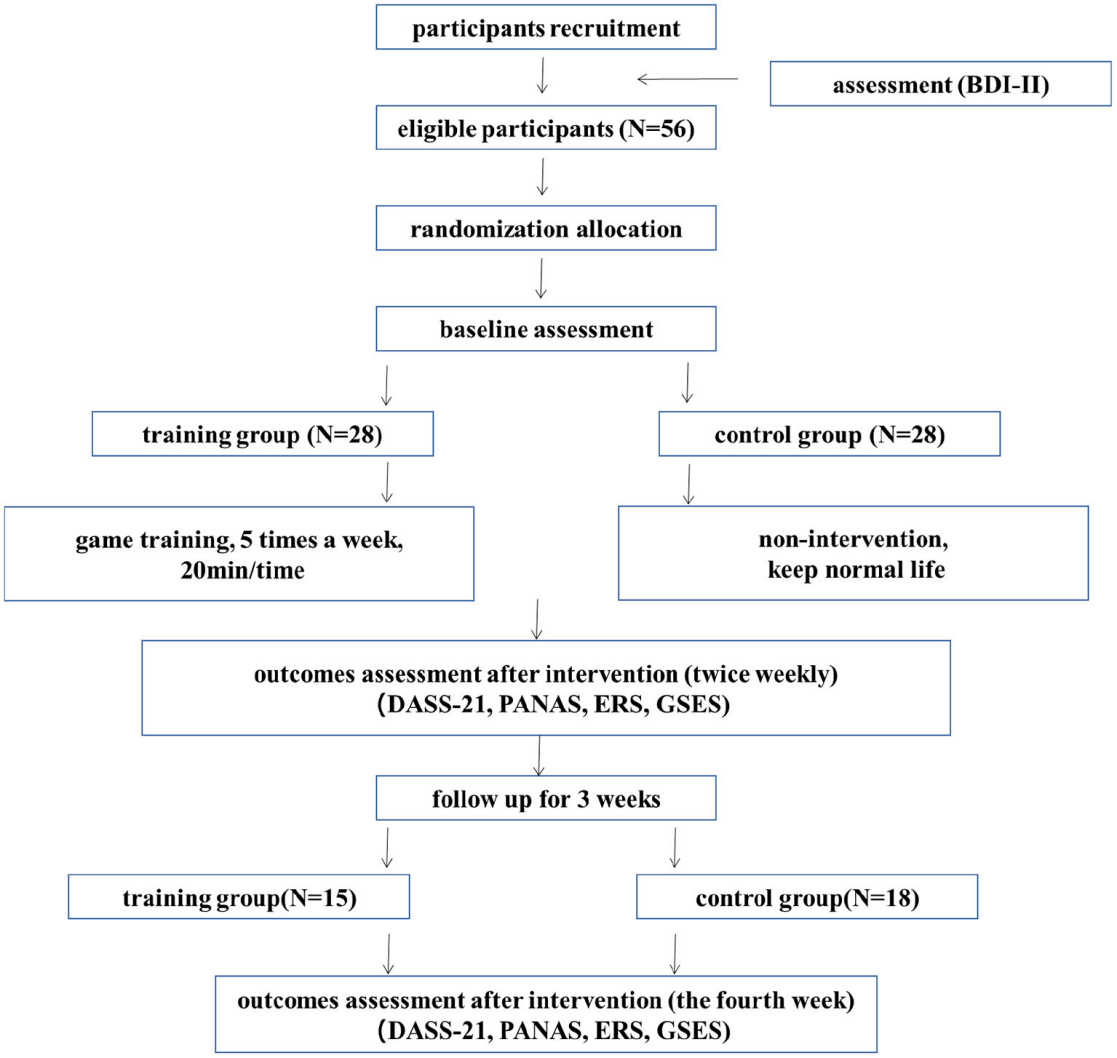
The study protocol.

Participants were recruited by distributing flyers, posters, and advertisements on social media and other online platforms. The interested participants were screened by research assistants based on the inclusion and exclusion criteria: we excluded participants that played music games for more than 5 h in the last 2 weeks to prevent the case of behavioral addiction and those diagnosed with psychotic disorders, major depression, or bipolar disorder. Participants with a score between 14 and 19 on the Beck Depression Inventory-II (BDI-II) scale were considered as subthreshold or mild depression (36, 37). Fifty-six participants who meet the criteria were included in the study. These participants received detailed information of the study and signed online informed consent. After recruitment, the participants were randomly assigned into the intervention group (28 participants, 18 females, age mean ± SD: 20.82 ± 2.31 years) or the control group (28 participants, 22 females, age mean ± SD: 20.67 ± 2.11 years). Baseline assessment of Depression Anxiety and Stress Scale (DASS-21) was then performed on all the participants. Afterwards, the experimental group underwent music-based casual video game training for 4 weeks. During the same time, the control group participants conducted daily life activities without any intervention. Few participants were unable to persevere and dropped out of the study during the 4 weeks of video game training or continued assessment. Finally, the experimental group left with 15 participants (12 females, age mean ± SD: 21.08 ± 2.40 years) and the control group left with 18 participants (14 females, age mean ± SD: 21.33 ± 3.18 years). The recruitment protocol is shown in Figure 1. All experimental protocols were approved by the institutional review board at the Sun Yat-sen University (2020-0515-0140).

### Music-based video game

**Figure 2:**
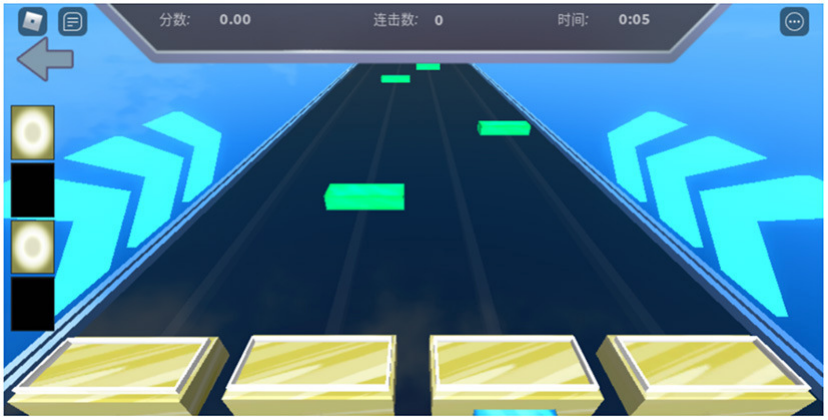
Representative interface of the musical video game.

The music-based video game used in this study was custom developed by Tencent (Shenzen, China) based on the requirement of this study and was implemented in the Roblox Platform. This game was easy-to-operate and could be adapted with different degrees of difficulty. The music for the present game is selected from previous intervention studies on affective disorders, e.g., depression (38) and anxiety (39). The interface of the game is shown in Figure 2. The study participants received a score based on their performance in operating the game at the right time according to the instructions appearing on the screen based on the rhythms of the background music. The scoring criteria of every press is listed in Table 1. Total score = [(perfect × 1) + (nice × 0.75) + (good × 0.6)] ÷ (perfect + nice + good + miss) × 100. Every participant was rated according to the total score that showed in Table 2.

**Table 1:** The scoring criteria of every press in the video game.

**Rating (key)**	**Reaction time**
PERFECT	−40 ~ 20 ms
NICE	20 ~ 50 ms
GOOD	50 ~150 ms
MISS	>150 ms

**Table 2:** Game rating corresponding to different total scores.

**Rating (association)**	**Total score**
SS	100–95
S	95–90
A	90–75
B	75–60
C	60–50
D	<50

### Beck depression inventory-II scoring system

The revised Chinese version of the Beck Depression Inventory-II (BDI-II) scale (36, 40) was used to assess the severity of depression. This measure is a reliable and valid measure of the depressive symptoms in both clinical and non-clinical populations (36). The BDI-II scale is comprised of 21 symptoms and features of depression, which are scored on a 4-point scale ranging from 0 to 3. The total score was calculated from the sum of scores for each of the 21 items. The standard cut-offs were as follows: 0–13, no depression; 14–19, mild depression; 20–28, moderate depression; and 29–63, severe depression. BDI (II) test showed high test-retest reliability (r = 0.55; *p* < 0.05) with a Cronbach's alpha value of 0.94 (40).

### Depression anxiety and stress scale (DASS-21)

The revised Chinese version of the Depression Anxiety and Stress Scale (DASS-21) (41, 42) was used in this study. This scale included 21 items with 7 items each for depression, anxiety, and stress. The cut-off values for depression were as follows: below 10, mild depression; 11–14, moderate depression, and 15–21, severe depression. The cut-off values for anxiety were as follows: 0–8, mild anxiety; 9–10, moderate anxiety; and 11–15, severe anxiety. The cut-off values for stress were as follows: 0–15, mild pressure; 16–19, moderate; and 20–26, severe pressure. The revised DASS-21test showed high test-retest reliability (r = 0.912) with a Cronbach's alpha value of 0.89 (41).

### Positive and negative affect scale

The revised Chinese version of PANAS (43) was used to assess the positive and negative emotions of the study participants. Both sections included 10 questions each. In this study, only the positive emotion scale was used to evaluate the participants. The progression of scores indicated the levels of negative or positive emotions. A score between 1 and 5 was awarded for each answer. The higher score indicated higher level of positive emotions. The PANAS test showed high test-retest reliability (r = 0.47) with a Cronbach's alpha value of 0.82 for the positive emotion (43).

### General self-efficacy scale

The revised Chinese version of the General Self-efficacy Scale (GSES) was used in this study (44, 45). This scale included 10 questions with the following four options: “completely incorrect,” “somewhat correct,” “mostly correct,” and “completely correct.” A score between 1 and 4 was awarded for each answer. The higher score indicated higher level of self-efficacy. The GSES test showed high test-retest reliability (r = 0.83) with a Cronbach's alpha value of 0.87.

### Emotion regulation questionnaire

The Chinese version of ERQ (46) consists of ten items, which are divided into two dimensions: cognitive reappraisal and expression inhibition. The measurement of cognitive reappraisal is composed of six items, and the measurement of expression inhibition is composed of four items. The emotional regulation scale is a seven-point scale, and the higher the score, the higher the frequency of using emotional regulation strategies. The retest reliability and Cronbach's alpha value of the emotion regulation questionnaire were 0.82 and 0.85, respectively. The retest reliability and Cronbach's alpha value of the expression inhibition dimension were 0.79 and 0.77, respectively.

### Study protocol

The study protocol is shown in Figure 1. The experimental group participants received 4 weeks of training for a minimum of 20 min per session, 5 sessions a week. The control group participants maintained their daily lifestyle. The DASS-21 were measured at baseline before the intervention. The DASS-21, PANAS, GESE, and ERQ were then measured twice weekly during the intervention.

### Statistical analysis

The statistical analysis was performed with the JASP software version 0.16.1.0 (JASP Team, 2022), which offers standard Bayesian statistical operations. Firstly, we performed two-sample *t-*test for the DAS total scores and D/A/S sub-dimension scores between the experimental and control groups during baseline before the intervention. Secondly, we performed two-way repeated-measures repeated measures analysis of variance (ANOVAs) with the group as the between-subject factor and the week as a dependent measure. Two-way repeated measures ANOVA was performed to test the differences between the groups and the weeks of intervention for the DAS scores, D/A/S aspects of the DAS scale and PA/ER/GSE scores. Thirdly, independent-sample *t*-tests were then performed to test differences between the experimental and control groups for each week. Lastly, to examine the correlation between DAS, PA, ER and GSE, we performed the Pearson correlation test between the DAS, PA, ER, and GSE scores measured over 4 weeks in the experimental group. We also report Bayesian Factor (BF) values for these analysis. Firstly, we analyzed the Bayesian two-sample *t*-test for the DAS total scores and D/A/S sub-dimension scores between the experimental and control groups during baseline before the intervention. Secondly, we analyzed the Bayesian ANOVA for these scores between the experimental and control groups during the 4 weeks, with the group as the between-subject factor and the week as a dependent measure. Bayesian repeated measures ANOVA was further performed to test the differences between the groups and the weeks of intervention for the depression, anxiety, and stress aspects of the DAS scale, respectively. Thirdly, we performed the Bayesian Pearson correlation test between the DAS, PA, ER, and GSE scores that were assessed during the 4 weeks in the experimental group. Fourthly, Bayesian analysis of covariance (ANCOVA) test was performed to further determine the relationship between the DAS, PA, ER, and GSE scores, with the subject as a random effect and the week as a fixed effect. When performing the Bayesian ANCOVA, we examined the best model among the various models by comparing the BFs. We used the default values for priors and other parameters in JASP. The evidence was evaluated to determine how the data fitted into the alternative model compared with the null model. The Bayes factor (BF) cutoffs were as follows: <3, anecdotal; 3–10, substantial; 10–30, strong; 30–100, very strong; and >100, extreme evidence (47).

## Results

### Music-based video game training for 4 weeks reduces DASS-21 scores in participants with subthreshold or mild depression

**Figure 3:**
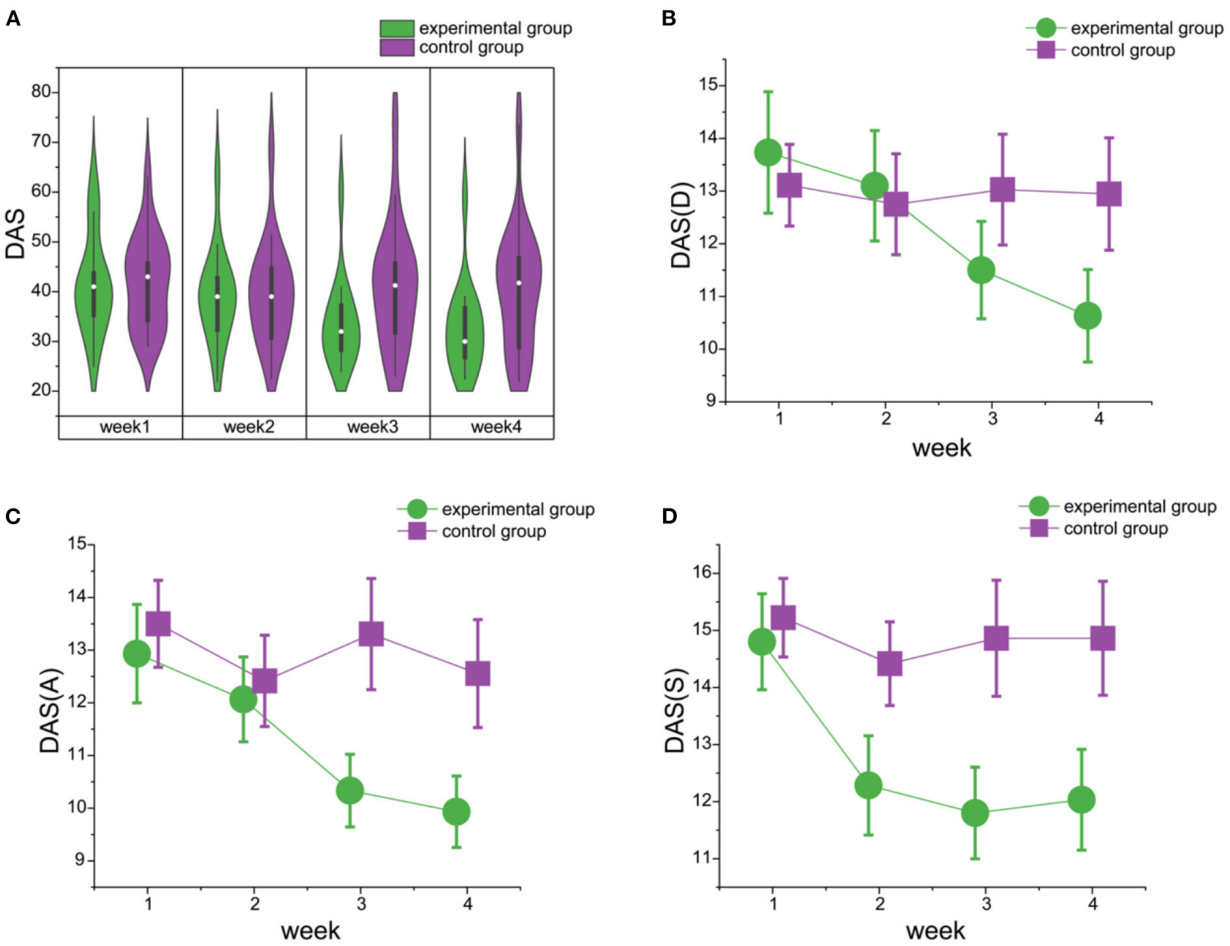
The changes in DAS scores during the four-week video game training in the experimental and control groups. **(A)** The violin plot shows the DAS scores for the study participants in the experimental (green) and the control (purple) groups during 4 weeks of video game training [experimental group: F_(3, 56)_ = 3.10, *p* = 0.03, $\eta_{\text{p}}^{2}$ = 0.14; BF_10_ = 3.306e+5; control group: F_(3, 68)_ = 0.18, *p* = 0.91, $\eta_{\text{p}}^{2}$ = 0.01; BF_10_ = 0.78). **(B)** The line chart shows the differences between the experimental and control groups on depression dimension scores. And the difference between the two groups for week 4 is supported by anecdotal evidence [week 4: t_(31)_ = −1.63, *p* = 0.06, Cohen's d = −0.57; BF_10_ = 1.67). **(C)** The line chart shows the differences between the experimental and control groups on anxiety dimension scores. And the difference between the two groups for weeks 3 and 4 is supported by substantial evidence [week 3: t_(31)_ = −2.26, *p* = 0.02, Cohen's d = −0.78; BF_10_ = 4.24; week 4: t_(31)_ = −2.04, *p* = 0.03, Cohen's d = −0.72; BF_10_ = 3.03]. **(D)** The line chart shows the differences between the experimental and control groups on stress dimension scores. And the difference between the two groups for weeks 3 and 4 is supported by substantial evidence [week 3: t_(31)_ = −2.48, *p* = 0.01, Cohen's d = −0.87; BF_10_ = 6.24; week 4: t_(31)_ = −2.15, *p* = 0.02, Cohen's d = −0.75; BF_10_ = 3.59]. DAS, Depression Anxiety Stress; D, Depression; A, Anxiety; S, Stress.

The study participants in the experimental and the control groups were matched in DASS-21 scores (mean ± SD: Experimental group 45.846 ± 9.36; Control group 42.61 ± 9.34; t_(29)_ = 0.95, *p* = 0.35, Cohen's d = 0.35; BF_10_ = 0.48). The baseline sub-scores were calculated for Depression (mean ± SD: Experimental group 15.08 ± 4.73; Control group 13.06 ± 3.54), Anxiety (mean ± SD: Experimental group 14.85 ± 3.29; Control group 13.28 ± 3.03), and Stress (mean ± SD: Experimental group 15.93 ± 3.15; Control group 16.28 ± 3.50). There was no significant difference between the experimental group and the training group in all the three sub-dimensions. (D: t_(29)_ = 1.36, *p* = 0.18, Cohen's d = 0.50, BF_10_ = 0.69; A: t_(29)_ = 1.37, *p* = 0.18, Cohen's d = 0.50, BF_10_ = 0.70; t_(29)_ = −0.29, *p* = 0.77, Cohen's d = −0.11, BF_10_ = 0.36). The baseline test indicated the participants were moderate in depression, severe in anxiety, mild to moderate in stress. The depression-anxiety-stress (DAS) scores of the experimental group gradually decreased during the 4 weeks of music-based video game training but the depression-anxiety-stress scores of the control group remained unchanged during the 4 weeks (see Table 3 and Figure 3).

**Table 3:** Assessment scores of the participants in the experimental and control groups during the 4 weeks of musicbased video game intervention.

**Assessment scales**	**Experimental group**				**Control group**			
	**1**	**2**	**3**	**4**	**1**	**2**	**3**	**4**
DAS	41.46 ± 9.98	39.10 ± 9.64	33.63 ± 8.82	32.60 ± 9.06	42.22 ± 9.04	39.58 ± 10.35	41.19 ± 12.62	40.36 ± 12.43
PA	27.80 ± 7.92	28.16 ± 6.41	28.78 ± 5.80	28.53 ± 6.93	25.00 ± 4.67	25.53 ± 4.80	24.25 ± 5.25	23.75 ± 5.70
ER	50.33 ± 7.58	50.60 ± 6.52	50.53 ± 6.89	50.57 ± 6.36	44.11 ± 6.06	43.64 ± 4.65	43.22 ± 5.36	43.31 ± 6.48
GSES	26.40 ± 3.31	27.17 ± 4.09	27.20 ± 4.56	26.80 ± 4.65	23.31 ± 3.55	23.31 ± 3.55	23.08 ± 4.69	23.25 ± 4.27

*DAS, Depression Anxiety and Stress Scale; PA, Positive Affect Scale; ER, Emotional Regulation Scale; GSES, General Self-Efficacy Scale; Weeks 1, 2, 3, and 4 are shown as 1, 2, 3, and 4, respectively*.

We performed a 2(GROUP: experimental group vs. control group)× 4 (WEEK: week 1, 2, 3, 4) classical and Bayesian repeated measures ANOVA for all the assessment scales to test differences between the study groups, as well as changes across weeks of intervention. For depression-anxiety-stress scales, the main effect of WEEK showed extreme evidence for the alternate hypothesis over the null hypothesis [F_(3,93)_ = 14.84, *p* < 0.01, $\eta_{\text{p}}^{2}$ = 0.33; BF_10_ = 1, 209.84]. *Post-hoc* tests showed extreme evidence for the alternative hypothesis with significant differences between the first and the fourth weeks of treatment [t_(32)_ = 6.16, *p* < 0.01, Cohen's d = 0.50; BF_10_ = 142.60]. The main effect of GROUP showed little evidence for the alternate hypothesis [F_(1,31)_ = 1.33, *p* = 0.26, $\eta_{\text{p}}^{2}$ = 0.04; BF_10_ = 0.73]. However, extreme evidence was observed for the interaction GROUP × WEEK [F_(3,93)_ = 12.39, *p* < 0.01, $\eta_{\text{p}}^{2}$ = 0.29; BF_10_ = 1.223e+7]. Bayesian independent-sample *t*-tests for the alternative hypothesis (experimental group<control group) demonstrated that evidence that the experimental group alleviated symptoms more than the control group increased over time [week 1: t_(31)_ = −0.11, *p* = 0.46, Cohen's d = −0.04; BF_10_ = 0.36; week 2: t_(31)_ = −0.14, *p* = 0.45, Cohen's d = −0.05; BF_10_=0.32; week 3: t_(31)_ = −1.96, *p* = 0.03, Cohen's d = −0.68; BF_10_ = 2.64; week 4: t_(31)_ = −2.01, *p* = 0.03, Cohen's d = −0.70; BF_10_ = 2.88]. Anecdotal evidence existed for the larger improvement in DAS symptoms for the experimental group in weeks 3 and 4.

**Figure 4:**
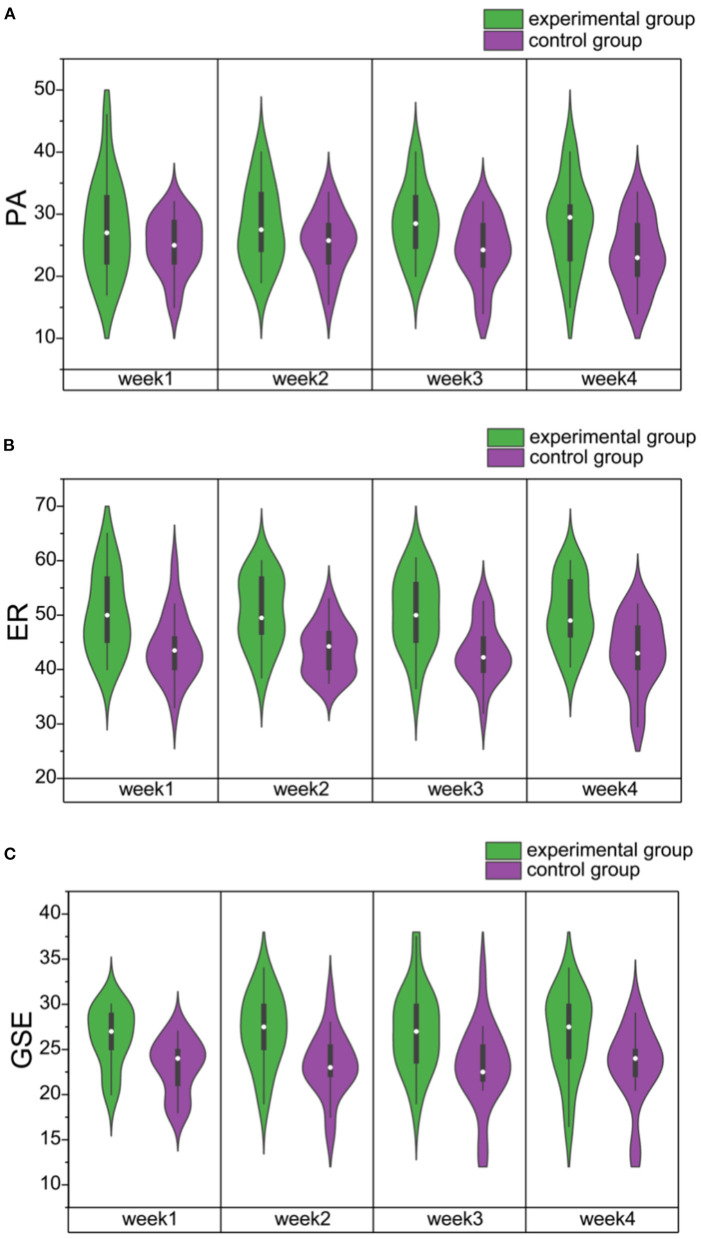
**(A)** The violin plots of PA scores for the experimental (green) and control (purple) groups during 4 weeks of video game training. **(B)** The violin plots of ER scores for the experimental (green) and control (purple) groups during 4 weeks of video game training. **(C)** The violin plots of GSE scores for the experimental (green) and control (purple) groups during 4 weeks of video game training. PA, Positive Affect; ER, emotional regulation; GSE, General Self-efficacy.

The positive affect (PA, see Figure 4A) scores showed anecdotal evidence for the main effect of GROUP [F_(1,31)_ = 3.75, *p* = 0.06, $\eta_{\text{p}}^{2}$ = 0.11; BF_10_ = 2.09] and no evidence for the main effect of WEEK [F_(3,93)_ = 0.37, *p* = 0.76, $\eta_{\text{p}}^{2}$ = 0.01; BF_10_ = 0.07] nor the interaction GROUP × WEEK [F_(3,93)_ = 1.36, *p* = 0.26, $\eta_{\text{p}}^{2}$ = 0.04; BF_10_ = 0.03].

The emotional regulation (ER, see Figure 4B) scores showed very strong evidence for the main effect of GROUP [F_(1,31)_ = 14.15, *p* < 0.01, $\eta_{\text{p}}^{2}$ = 0.31; BF_10_ = 39.97] but no evidence for the main effect of WEEK [F_(3,93)_ = 0.08, *p* = 0.97, $\eta_{\text{p}}^{2}$ = 0.01; BF_10_ = 0.05] nor the interaction GROUP × WEEK [F_(3,93)_ = 0.18, *p* = 0.91, $\eta_{\text{p}}^{2}$ = 0.01; BF_10_ = 0.17]. The *post-hoc* tests showed extreme evidence for the alternative hypothesis of significant differences between the experimental and the control groups [t_(32_= 3.76, *p* < 0.01, Cohen's d = 1.14; BF_10_=1.398e+7], but the differences between the first and the fourth weeks were not significant [t_(32)_ = 0.38, *p* = 1.00, Cohen's d = 0.05; BF_10_ = 0.20].

The general self-efficacy (GSE, see Figure 4C) scores showed substantial evidence for the main effect of GROUP [F_(1,31)_ = 8.75, *p* = 0.01, $\eta_{\text{p}}^{2}$ = 0.22; BF_10_ = 7.08], but no evidence for the main effect of WEEK [F_(3,93)_ = 0.30, *p* = 0.83, $\eta_{\text{p}}^{2}$ = 0.01; BF_10_ = 0.06] nor the interaction GROUP × WEEK [F_(3,93)_ = 0.23, *p* = 0.88, $\eta_{\text{p}}^{2}$ = 0.01; BF_10_ = 0.04). *Post-hoc* tests showed extreme evidence for the alternative hypothesis of significant differences between the experimental and the control groups [t_(32)_ = 2.96, *p* < 0.01, Cohen's d = 0.92; BF_10_ = 33,847.20). However, the differences between the first and the fourth weeks showed little evidence to support the alternate hypothesis [t_(32)_ = −0.50, *p* = 1.00, Cohen's d = −0.07; BF_10_ = 0.202).

### Music-based video games training decreases depression, anxiety, and stress scores as a function of the weeks of intervention

Furthermore, a 2(GROUP: experimental group vs. control group)× 4 (WEEK: week 1, 2, 3, 4) Bayesian repeated measures ANOVA was performed for the depression, anxiety, or stress aspects of the DAS scale respectively to test the differences between the groups, and changes across the weeks of intervention. The depression scores (D) showed strong evidence for the main effect of WEEK [F_(3,93)_ = 9.28, *p* = <0.01, $\eta_{\text{p}}^{2}$ = 0.23; BF_10_ = 39.693], but little evidence for the main effect of GROUP [F_(1,31)_ = 0.28, *p* = 0.60, $\eta_{\text{p}}^{2}$ = 0.01; BF_10_ = 0.61]. However, extreme evidence was observed for the interaction GROUP × WEEK [F_(3,93)_ = 9.16, *p* = <0.01, $\eta_{\text{p}}^{2}$ = 0.23; BF_10_ = 18,159.24]. *Post-hoc* tests showed strong evidence for the alternative hypothesis because of significant differences between the first and the fourth weeks [t_(32)_ = 4.89, *p* < 0.01, Cohen's d = 0.41; BF_10_ = 10.06], but there was little evidence to support the alternate hypothesis based on the differences between the experimental and the control groups [t_(32)_ = −0.53, *p* = 0.60, Cohen's d = −0.18; BF_10_ = 0.30]. Bayesian independent-sample *t*-tests for the alternative hypothesis (experimental group<control group) demonstrated that evidence that the experimental group alleviated depression symptoms more than the control group increased over time [week 1: t_(31)_ = 0.46, *p* = 0.68, Cohen's d = 0.17; BF_10_ = 0.25; week 2: t_(31)_ = 0.24, *p* = 0.60, Cohen's d = 0.09; BF_10_ = 0.28; week 3: t_(31)_ = −1.07, *p* = 0.14, Cohen's d = −0.37; BF_10_ = 0.85; week 4: t_(31)_ = −1.63, *p* = 0.06, Cohen's d = −0.57; BF_10_ = 1.67]. Anecdotal evidence existed for the larger improvement in depression symptoms for the experimental group in week 4.

The anxiety scores (A) showed extreme evidence for the main effect of WEEK [F_(3,93)_= 13.47, *p* = <0.01, $\eta_{\text{p}}^{2}$ = 0.31; BF_10_ = 1,532.08], but little evidence for the main effect of GROUP [F_(1,31)_ = 1.84, *p* = 0.19, $\eta_{\text{p}}^{2}$ = 0.06; BF_10_ = 0.84]. However, the interaction between the group and the week showed extreme evidence for the alternate hypothesis [F_(3,93)_ = 9.02, *p* = <0.01, $\eta_{\text{p}}^{2}$ = 0.23;BF_10_ = 7.556e+5). *Post-hoc* tests showed strong evidence for the alternative hypothesis of significant differences between the first and the fourth weeks [t_(32)_ = 6.16, *p* < 0.01, Cohen's d = 0.55; BF_10_ = 311.72] and substantial evidence for the alternative hypothesis of differences between the experimental and the control groups [t_(32)_ = − 1.36, *p* = 0.19, Cohen's d = −0.45; BF_10_ = 3.52]. Bayesian independent-sample *t*-tests for the alternative hypothesis (experimental group<control group) demonstrated that evidence that the experimental group alleviated symptoms more than the control group increased over time [week 1: t_(31)_ = −0.46, *p* = 0.33, Cohen's d = −0.16; BF_10_ = 0.47; week 2: t_(31)_ = −0.29, *p* = 0.39, Cohen's d = −0.10; BF_10_ = 0.41; week 3: t_(31)_ = −2.26, *p* = 0.02, Cohen's d = −0.79; BF_10_ = 4.24; week 4: t_(31)_ = −2.04, *p* = 0.03, Cohen's d = −0.72; BF_10_ = 3.03). Substantial evidence existed for the larger improvement in anxiety symptoms for the experimental group in weeks 3 and 4.

The stress scores (S) showed substantial evidence for the main effect of WEEK [F_(3,93)_ = 6.29, *p* = <0.01, $\eta_{\text{p}}^{2}$ = 0.17; BF_10_ = 7.236] and anecdotal evidence for the main effect of GROUP [F_(1,31)_ = 2.79, *p* = 0.11, $\eta_{\text{p}}^{2}$ = 0.08; BF_10_ = 1.11]. *Post-hoc* tests showed strong evidence for the alternative hypothesis of significant differences between the experimental and the control groups [t_(32)_ = −1.67, *p* = 0.11, Cohen's d = −0.53; BF_10_ = 10.83], and substantial evidence for the alternative hypothesis based on the differences between the first and the fourth weeks [t_(32)_ = 3.67, *p* = 0.01, Cohen's d = 0.43; BF_10_ = 5.99]. Bayesian independent-sample *t-*tests for the alternative hypothesis (experimental group<control group) showed that evidence that the experimental group alleviated symptoms more than the control group increased over time [week 1: t_(31)_ = −0.39, *p* = 0.35, Cohen's d = −0.14; BF10 = 0.45; week 2: t_(31)_ = −0.73, *p* = 0.24, Cohen's d = −0.26; BF_10_ = 0.60; week 3: t_(31)_ = −2.48, *p* = 0.01, Cohen's d = −0.87; BF_10_ = 6.24; week 4: t_(31)_ = −2.15, *p* = 0.02, Cohen's d = −0.75; BF_10_ = 3.59). Substantial evidence existed for the larger improvement in stress symptoms for the experimental group in weeks 3 and 4.

### DAS score correlates negatively with general self-efficacy, positive emotions, and emotional regulation

Bayesian Pearson correlation analysis demonstrated association between all the four assessment scales across all the participants in the experimental group and the weeks (Table 4). There was extreme evidence for a positive correlation between GSE and ER (r = 0.605, *p* < 0.01, BF_10_ = 5.857e+4,) and a negative correlation between DAS and PA (r = −0.528, *p* < 0.01, BF_10_ = 1,527.176). Moreover, there was strong evidence for a positive correlation between GSE and PA (r = 0.420, *p* < 0.01, BF_10_ = 36.878) and a negative correlation between GSE and DAS (r = −0.408, *p* < 0.01, BF_10_ = 26.638). Furthermore, there was substantial evidence for a negative correlation between DAS and ER (r = −0.343, *p* < 0.01, BF_10_ = 5.452) and anecdotal evidence for a positive correlation between ER and PA (r = 0.298, *p* = 0.02, BF_10_ = 2.206).

**Table 4:** Bayesian pearson correlations between DAS, PA, ER, and GSE scores.

**Variables**		**DAS**	**PA**	**ER**	**GSE**
DAS	Pearson's r	—			
	BF10	—			
PA	Pearson's r	−0.528	—		
	BF10	1,527.176	—		
ER	Pearson's r	−0.343	0.298	—	
	BF10	5.452	2.206	—	
GSE	Pearson's r	−0.408	0.420	0.605	—
	BF10	26.638	36.878	58,568.231	—

**Figure 5:**
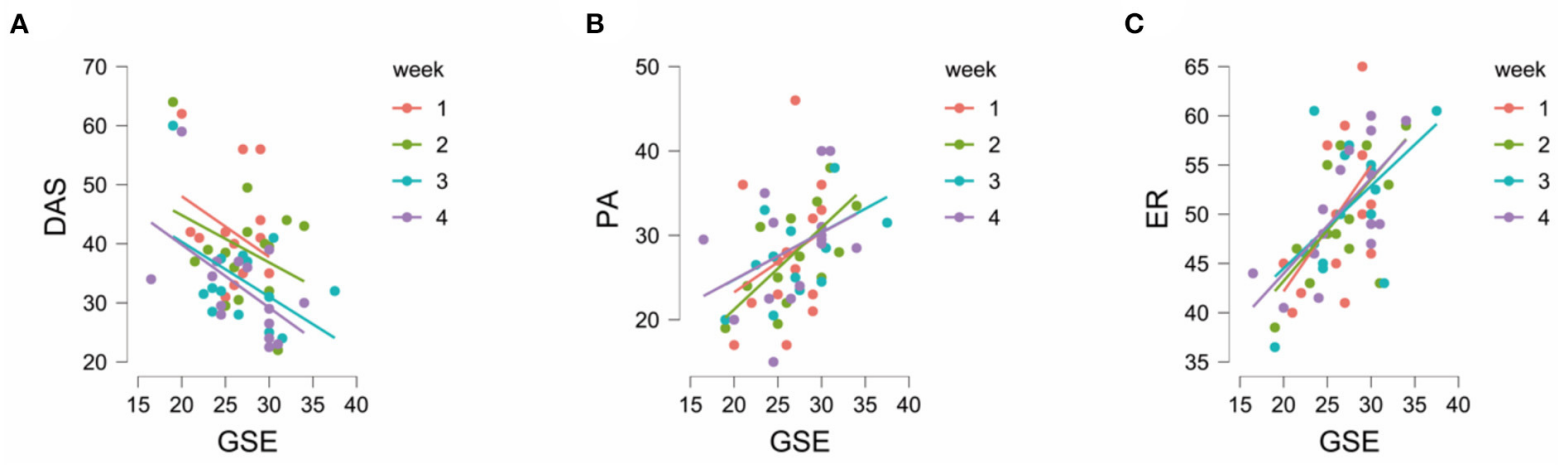
**(A)** The ANCOVA Correlation plots for GSES vs. DAS. **(B)** The ANCOVA Correlation plots for GSES vs. PA. **(C)** The ANCOVA Correlation plots for GSES vs. ER. DAS, Depression Anxiety Stress; PA, Positive Affect; ER, Emotional Regulation; GSES, General Self-Efficacy Score.

We then performed Bayesian ANCOVA to test the relationship between the four scales in the experimental group, with PARTICIPANT as a random effect and WEEK as a fixed effect. When DAS was considered as the dependent variable and the other three scales were considered as the covariates, the mixed effects model with WEEK and GSES was the best model (BF_10_ = 5.2946e+5 compared with the null model with only the subject). The 95% credible interval for the 4 weeks was provided by the reconstructed posterior model [week 1 (2.521, 6.015); week 2 (0.617, 3.884); week 3 (−4.553, −1.104); week 4 (−5.592, −2.125) and SE (−0.893, 0.123), Note the changes across weeks]. The ANCOVA correlation plots were visualized to demonstrate the mixed effects of week and GSES (Figure 5A). The slope of the graph was stable but the intercepts of the four lines decreased over weeks. This suggested that self-efficacy influenced depression, anxiety, and stress in the study participants across weeks.

Bayesian ANCOVA was also performed to evaluate the effects of general self-efficacy over the emotional assessment. When PA was considered as the dependent variable and the other three scales were considered as the covariates, the best model was the one with the main effects of GSE (BF_10_ = 59.06 compared with the null model with the subject only). We observed strong evidence for the effects of GSES on PA (BF_incl_ = 9.73). When emotional regulation was considered as the dependent variable and the other three scales were considered as the covariates, the model with the main effects of GSE was again the best model [BF_10_ = 2,371.12 compared to the null model with only the subject]. There was extreme evidence for GSE affecting ER (BF_incl_ = 450.88). The ANCOVA correlation plots for GSES vs. PA and GSES vs. ER are shown in Figures 5B,C, respectively.

## Discussion

This study demonstrated that 4 weeks of music-based video game training significantly alleviated anxiety, stress, and depression, as well as the total DAS scores in the experimental participants with subthreshold or mild depression. In contrast, the matched control group participants with subthreshold or mild depression did not show any significant changes in all the assessment during these 4 weeks. Moreover, the differences between the experimental and the control groups increased incrementally over the weeks of training. These results demonstrated the effectiveness of the music-based video game training program used in the study. General linear model analysis showed that the changes in DAS scores were dependent on the number of training weeks and the status of self-efficacy. This suggested that the music-based video game training significantly improved the cognitive functions, and then alleviated depression symptoms. Furthermore, improvements in self-efficacy positively affected the PA and ER scores, thereby suggesting that positive cognitive changes in response to music-based video game training led to better emotional states and emotional regulations.

Prior work has proved that casual video games improved the emotional state of individuals (48). In addition, video games with short duration and lower cognitive demands, such as Tetris, decrease the stress levels and improve the mood of players during the game resulting in physiological changes such as reduced heart rate variability and altered brain waves (49). The prefrontal lobe activity was significantly increased in the healthy elderly participants that underwent 1 month of video game training along with changes in the biomarkers associated with the antidepressant response (50). The present study further demonstrated that when music elements were included, our custom-designed music-based casual video game training significantly decreased the depression, anxiety, and stress scores in the training group.

However, previous studies did not reveal the mechanisms how casual video game training changed emotional states. Our results further demonstrated that music-based video games without high cognitive needs improved the depression, anxiety, and stress levels of the study participants with subthreshold or mild depression after only 1 month playing, by changing the cognitive state and improving positive emotions and the ability to regulate emotions.

We also found a negative correlation between DAS scores and self-efficacy. This result was consistent with the study of Bandura (51) that reported a relationship between negative emotions as well as depression and lower level of self-efficacy or outcome expectations. In addition, depression was found negatively correlated with induced self-efficacy (when the participants were told that their ability was high or low) (52). Our results demonstrate that music-based video game training can improve the emotional state of participants with subthreshold or mild depression by enhancing self-efficacy. In this music-based video game, the difficulty level was adjusted according to the skills of the study participants. For example, if the participant could hit the drop key correctly, the difficulty level of the game increased automatically and required greater attention and faster response speed from the player. When the hit rate of the player decreased, the difficulty levels of the game were automatically reduced. Moreover, the players were provided with immediate feedback. This process gave the participants a sense of control over the game, thereby improving their self-efficacy and reducing their negative emotions such as anxiety, depression, and stress.

The music used in this study has been widely used in multiple studies focused on affective disorders [depression (38) and anxiety (39)], ranging from popular to classical piano music.

Positive Emission Tomography (PET) studies demonstrated that music altered the brain regions associated with reward and emotions (53). Music increased resting-state cerebral blood flow (rCBF) in the left ventral striatum and dorsomedial midbrain, and decreased rCBF in the right amygdala, left hippocampal amygdala, and ventromedial prefrontal lobe (53). The activity of these brain structures was associated with reward and stimuli related to food, sex, and drug abuse (53). Therefore, the study participants enjoyed beautiful music during music-based video game training, which probably affected the brain areas related to pleasant emotions. This potentially suppressed the negative emotional states such as anxiety, depression, and stress and improved positive emotions.

During video game training, participants sometimes experienced positive emotions called “flow,” which included being intensely focused on the task in hand, coordination of action and consciousness, and a feeling of being in control of one's actions (54). This increased the positive emotions (55) and reduced the anxiety levels (56). The sense of control generated by “flow” improved self-efficacy of the subthreshold or mild depression participants and was positively associated with positive emotions (20). This “flow” experience coincides with our findings that music-based video game training increases self-efficacy and subsequently improves the positive emotions of the study participants and reduces their depression, anxiety, and stress.

Previous studies demonstrated that music could further activated the areas of cognitive monitoring and emotional regulation in the brain (53, 57). The Music-based Emotional Regulation (MBE) theory for the elderly suggests that music improves emotional regulation by diverting the attention of the individual from negative events (58). Meanwhile, video games also improved emotional regulation (16) by enhancing cognitive control (15). Therefore, our protocol combining music and video game training successfully reduced depression, increased positive emotions and improved emotional regulation. On the contrary, emotional regulation is altered in individuals experiencing highly negative emotions (59). The protocol used in our experiment could potentially be beneficial for them.

It has been suggested that individuals with low self-efficacy were unable to adjust their emotions (60–62). Interestingly, our study demonstrated a positive correlation between self-efficacy and emotional regulation. Music-based video game training here increased self-efficacy, which could further affect cognitive reappraisal. Individuals with high self-efficacy are confident in their ability to overcome difficulties (63). This perception encourages them to solve problems effectively and reduces negative emotions such as depression, anxiety, and stress (22, 63).

To our knowledge, this is the first study to investigate the beneficial effect of music-based casual video game on reducing depression levels in young participants with subthreshold or mild depression. Previous games with music have mostly used music as background music. However, music in our study is set as a target what participants need to respond to. So, the engagement of participants in the music would be more profound. Classifying the degree of depression is debatable because of the existence of several states between the non-depressed state to the extremely depressed state (5, 64, 65). Mild or subthreshold depression significantly increases the risk of major depression in adolescents and adults (64, 66). Thus, timely treatment of depression is important. In comparison with the traditional treatments for depression, music games on mobile phones are portable, low-cost, and not limited by space and time.

This study has a few limitations. Firstly, although this study was pre-registered, and the study participants were recruited accordingly, as experiment progressed, several participants dropped off from the study, thereby reducing the sample size. The participants may have dropped out for the following reasons: (1) This game was a newly developed video game, and the amount of music (Strict Copyright investigation in Roblox platform) available to the players was not many (20 pieces of music for the first version). The visual presentation of this game is simple, and the player could not choose the game background, button shape, and others. Therefore, the game may have been boring during the month-long training period. Secondly, most of the recruited participants were college students. This may have resulted in sample bias. Finally, this study only focused on the behavioral results. In the future, neuroimaging technologies could be used to explore the neural mechanisms underlying the changes in depression, anxiety, and stress. Previous studies explored the effects of video games on brain plasticity in the elderly and adolescents. Thus, our findings might also be extended to different age groups.

In conclusion, our results demonstrated that training for 4 weeks with music-based casual video games promoted positive emotions and emotional regulation by improving self-efficacy, thereby decreasing depression, anxiety, and stress levels.

## Data availability statement

The raw data supporting the conclusions of this article will be made available under reasonable request to the corresponding author YK.

## Ethics statement

The studies involving human participants were reviewed and approved by the Institutional Review Board at the Sun Yat-sen University. The patients/participants provided their written informed consent to participate in this study.

## Author contributions

YK and TQ conceived and supervised the study. YK, XL, and LN designed the experiment. XL, MZ, and YZ carried out the experiment. YK and YZ analyzed the data. MZ, YZ, and YW drafted the first manuscript. All authors contributed to the revision of the manuscript.

## Funding

This work was supported by funding by the National Natural Science Foundation of China (Grant No. 32171082), the National Social Science Foundation of China (Grant No. 17ZDA323), and the Shanghai Committee of Science and Technology (Grant No. 19ZR1416700) to YK.

## Conflict of interest

Author TQ was employed by Tencent Healthcare. The remaining authors declare that the research was conducted in the absence of any commercial or financial relationships that could be construed as a potential conflict of interest.

## Publisher's note

All claims expressed in this article are solely those of the authors and do not necessarily represent those of their affiliated organizations, or those of the publisher, the editors and the reviewers. Any product that may be evaluated in this article, or claim that may be made by its manufacturer, is not guaranteed or endorsed by the publisher.
